# Colonic epithelial cell-specific TFEB activation: a key mechanism promoting anti-bacterial defense in response to *Salmonella* infection

**DOI:** 10.3389/fmicb.2024.1369471

**Published:** 2024-04-22

**Authors:** Shanshan Rao, Pu Huang, Yi-Yu Qian, Yu Xia, Hongfeng Zhang

**Affiliations:** ^1^Department of Pathology, the Central Hospital of Wuhan, Tongji Medical College, Huazhong University of Science and Technology, Wuhan, China; ^2^Department of Obstetrics and Gynecology, Shandong Provincial Hospital Affiliated to Shandong First Medical University, Jinan, China; ^3^Cancer Biology Research Center (Key Laboratory of the Ministry of Education, Hubei Provincial Key Laboratory of Tumor Invasion and Metastasis), Tongji Hospital, Tongji Medical College, Huazhong University of Science and Technology, Wuhan, China; ^4^National Clinical Research Center for Obstetrics and Gynecology, Department of Gynecological Oncology, Tongji Hospital, Tongji Medical College, Huazhong University of Science and Technology, Wuhan, China

**Keywords:** colitis, colonic epithelial cells, TFEB, *Salmonella*, anti-bacterial peptides

## Abstract

Colitis caused by infections, especially *Salmonella*, has long been a common disease, underscoring the urgency to understand its intricate pathogenicity in colonic tissues for the development of effective anti-bacterial approaches. Of note, colonic epithelial cells, which form the first line of defense against bacteria, have received less attention, and the cross-talk between epithelial cells and bacteria requires further exploration. In this study, we revealed that the critical anti-bacterial effector, TFEB, was primarily located in colonic epithelial cells rather than macrophages. *Salmonella*-derived LPS significantly promoted the expression and nuclear translocation of TFEB in colonic epithelial cells by inactivating the mTOR signaling pathway *in vitro*, and this enhanced nuclear translocation of TFEB was also confirmed in a *Salmonella*-infected mouse model. Further investigation uncovered that the infection-activated TFEB contributed to the augmentation of anti-bacterial peptide expression without affecting the intact structure of the colonic epithelium or inflammatory cytokine expression. Our findings identify the preferential distribution of TFEB in colonic epithelial cells, where TFEB can be activated by infection to enhance anti-bacterial peptide expression, holding promising implications for the advancement of anti-bacterial therapeutics.

## Introduction

Intestinal epithelial cells play a pivotal role in the physiological processes of the entire organism through nutrient absorption and bowel homeostasis (Parikh et al., 2019). Due to the fact that thousands of microorganisms, including probiotics and malignant microbes, survive in the gastrointestinal lumen, epithelial cells function as the first barrier to keep the probiotics alive and defend against pathogenic bacteria (Groschwitz and Hogan, 2009; Antoni et al., 2014; Rogers et al., 2021). Once the homeostasis is broken up, the intestine and colon might be afflicted by inflammatory bowel disease (IBD) or colitis (Rogers et al., 2021). Invasive bacterial infection is a major contributor to colitis. In addition to *Campylobacter jejuni*, *Shigella*, *Yersinia enterocolitica*, *Clostridium difficile*, and *Mycobacterium tuberculosis*, *Salmonella* is one of the most common bacteria responsible for infectious colitis (Azer and Sun, 2021). However, the cross-talk between epithelial cells and *Salmonella* is still not fully understood.

The colonic epithelium is composed of monolayer cells that are characterized by tightly arranged apical-lateral membrane junctions. Through desmosomes, adherens junctions, and tight junctions, these cellular units form an intact screen that separates the external microbiota from the internal organism (Groschwitz and Hogan, 2009). Additionally, epithelial cells secrete anti-bacterial peptides, cytokines, and mucus to fight against bacterial invasion (Groschwitz and Hogan, 2009; Antoni et al., 2014). However, many malignant pathogens overcome the line of defense established by the epithelium, leading to further infection.

*Salmonella*, a Gram-negative bacterium, is a highly pathogenic bacterium that invades enterocytes via fimbrial adhesins and the *Salmonella* pathogenicity island 1 (SPI1)-encoded type III secretion system (T3SS) (Tahoun et al., 2012; Lorkowski et al., 2014; Gul et al., 2023). Intracellular *Salmonella* is wrapped up in vacuoles known as *Salmonella*-containing vacuoles (SCVs) for better replication and escape from destruction (Birmingham et al., 2006; Hautefort et al., 2008). Xenophagy plays a major role in the defense of intracellular *Salmonella*, 20% of which co-localizes with LC3-positive autophagosomes within 1 h of infection (Birmingham et al., 2006; Bauckman et al., 2015). For further degradation, *Salmonella*-containing autophagosomes fuse with lysosomes. Almost 60 kinds of acid hydrolases, including lipases, proteases, glycosidases, and acid phosphatases, are present in lysosomes, where they play a crucial role in digestion (Perera and Zoncu, 2016; Ballabio and Bonifacino, 2020). Atg5-deficient mouse embryonic fibroblast indulges *Salmonella* growth in the cytosol (Birmingham et al., 2006).

TFEB is viewed as a critical transcription factor (TF) that dominates the expression of autophagic and lysosomal proteins (Settembre et al., 2011; Napolitano and Ballabio, 2016; Panwar et al., 2023; Xia et al., 2023). Because of the important role of the autophagy-lysosome in the degradation of bacteria, TFEB contributes to the defense against microbial infection. Emerging evidence verified that reinvigorating the activity of TFEB did enhance the restriction of *Salmonella* replication (Ammanathan et al., 2019; Schuster et al., 2022). Our previous findings also proved that *Salmonella* escaped degradation by suppressing TFEB in bone marrow-derived macrophages (BMDMs) (Rao et al., 2020). Additionally, TFEB was also demonstrated to modulate the expression of pro-inflammatory cytokines in RAW264.7 cells in the presence of *Escherichia coli* (Visvikis et al., 2014). Therefore, how TFEB is regulated under the infection of *Salmonella* and what the distinctive functions of TFEB are in colonic epithelial cells are still ambiguous.

In this study, we clarified that TFEB was predominantly distributed in the epithelium cells of the colon rather than other cells. *Salmonella*-derived LPS enhanced autophagic and lysosomal gene expression by enhancing the activity of TFEB. Furthermore, TFEB was translocated to the nucleus in colonic enterocytes after *Salmonella* infection in C57/B6 mice. Finally, we found that TFEB positively regulated several anti-bacterial peptides that contributed to the defense against *Salmonella*. Together, we unveil the critical role of TFEB in epithelial cells against *Salmonella* infection.

## Results

### TFEB primarily distributes in enterocytes in mice’s colon

**Figure 1:**
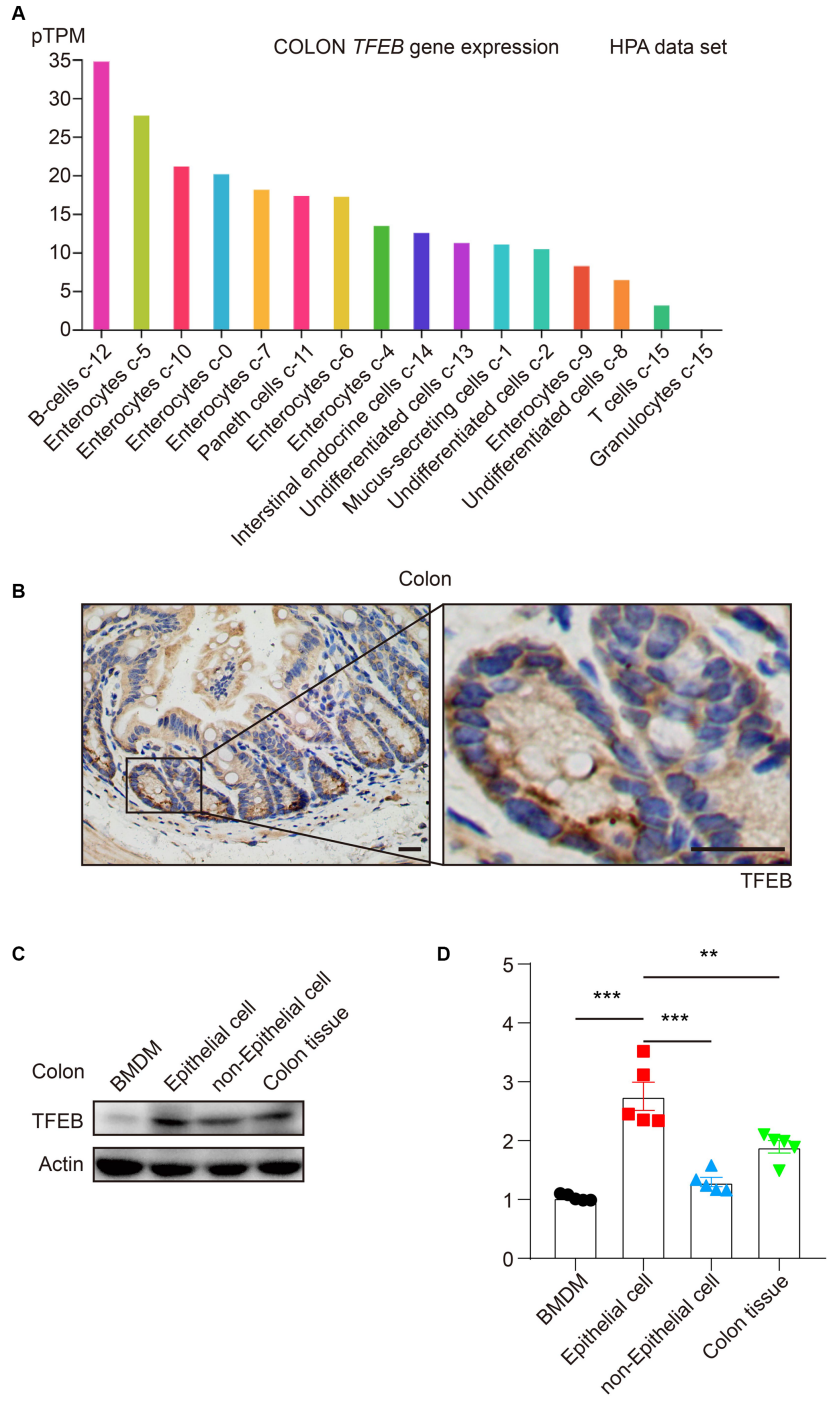
TFEB predominantly expresses in colonic epithelial cells. **(A)** The histogram presents TFEB expression in the colon according to the HPA data set. **(B)** Representative IHC graphs exhibit TFEB staining in a murine healthy colon. Scale bars: 20 μm. **(C,D)** A healthy murine colon was divided into epithelium, colon tissue without epithelium, and entire colon tissue. Determining the TFEB and actin in BMDM and colon tissue (*n* = 5) as indicated. Representative Western blotting bands are shown **(C)** and quantified with ImageJ **(D)**. Mean values ± s.e.m. ***p < 0.001 using the one-way ANOVA with Dunnett’s test in **(D)**.

Because TFEB plays an essential role in the colon under bacterial infection and the majority of research focuses on macrophages (Gray et al., 2016; El-Houjeiri et al., 2019; Rao et al., 2020; Schuster et al., 2022), we were eager to explore the detailed distribution of TFEB, which might clarify the TFEB-mediated anti-bacterial responses. According to the Human Protein Atlas (HPA), TFEB is highly expressed in enterocytes (Figure 1A). To further verify this finding, the colon tissues of healthy C57/B6 mice were dissected and subjected to immunohistochemical (IHC) staining to determine the expression of TFEB (Figure 1B). The results were consistent with the dataset. In addition, the primary colonic epithelial cells of mice were isolated and used to examine the protein levels of TFEB, in comparison with BMDM, colon tissue without epithelium, and whole colon tissue. Western blotting analysis showed that TFEB was less expressed in the macrophages and more expressed in the epithelial cells (Figures 1C,D). Therefore, the epithelium may be the cardinal battlefield for TFEB to exert its anti-bacterial functions.

### LPS boosts the expression of TFEB and autophagy-lysosome proteins

**Figure 2:**
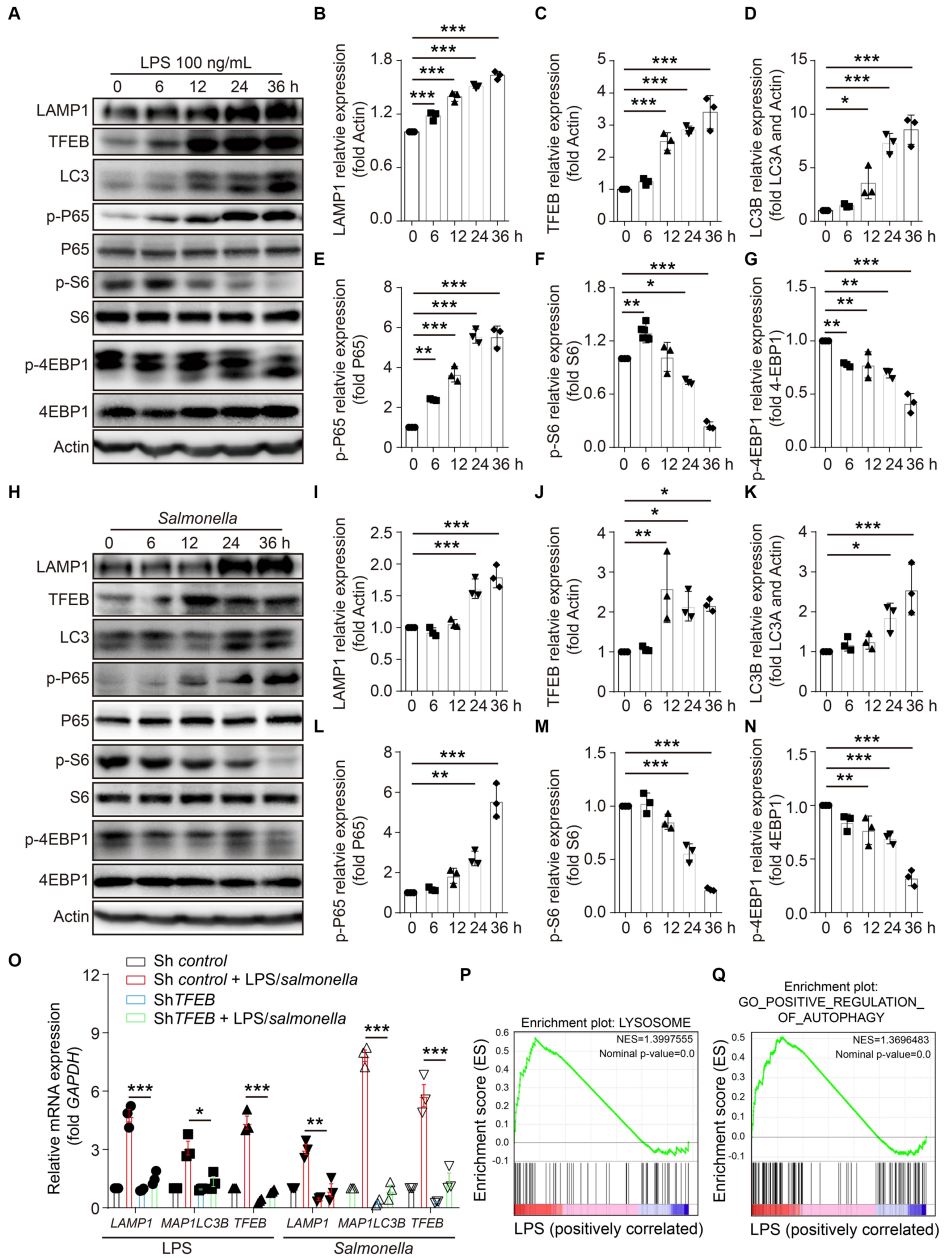
LPS enhances the expression of TFEB and autophagy-lysosome-related proteins in colonic epithelial cells. **(A–G)** Human colonic epithelium cell line Caco 2 cells were stimulated with 100 ng/mL of LPS for 0 h, 6 h, 12 h, 24 h, or 36 h. Western blotting was used to detect the protein level of LAMP1, TFEB, LC3, actin, phosphorylation, and total P-65, S6, and 4EBP1 **(A)**. Histograms exhibit the statistical analyses **(B–G)**. **(H–N)** Caco 2 cells were infected with *Salmonella* (MOI = 2) for 0 h, 6 h, 12 h, 24 h, or 36 h. Western blotting was used to detect the protein level of LAMP1, TFEB, LC3, actin, and the phosphorylation and total protein of P-65, S6, and 4EBP1 **(H)**. Histograms exhibit the statistical analyses **(I–N)**. **(O)** Control and sh*TFEB* Caco 2 cells were treated with LPS (100 ng/mL) or infected with *Salmonella* (MOI = 2) for 24 h. The mRNA levels of *LAMP1*, *MAP1LC3B*, and *TFEB* were determined by RT-PCR. **(P)** Lysosome gene set, Z-score values, and enrichment plot after GSEA analysis between LPS-treated and control HT29 cells (NES = 1.3997555, *p* < 0.01, GSE113581). **(Q)** Autophagy gene set, Z-score values, and enrichment plot after GSEA analysis between LPS-treated and control HT29 cells (NES = 1.3696483, *p* < 0.01, GSE113581). Representative bands are from three independent experiments. Mean values ± s.e.m. *p < 0.05, **p < 0.01, ***p < 0.001 using the one-way ANOVA with Dunnett’s test in **(B–G)**, **(I–N)**, and **(O)**. The permutation test was used in GSEA.

To investigate the effects of infection on the regulation of TFEB expression and functions, LPS derived from *S.typhimurium* was used in the stimulation of a colonic epithelial cell line Caco 2 for 0 h, 6 h, 12 h, 24 h, and 36 h. TFEB, the lysosomal marker LAMP1, and the autophagic molecule LC3 were determined in these treated cells using Western blotting. The results revealed that LPS enhanced the expression of all the determined proteins in a time-dependent manner (Figures 2A–D). NF-κB and mTOCR signaling pathways usually function as the two major responders to the stimulation of LPS (Temiz-Resitoglu et al., 2017; Lund et al., 2022). Subsequently, to figure out the potential mechanisms underlying the increase of TFEB, the phosphorylation of NF-κB and mTOCR1 signaling pathways (S6 and 4EBP1) was detected at different time points. Interestingly, the activity of P65 was remarkably potentiated by LPS, indicating that epithelial cells underwent cellular responses to LPS. The levels of p-S6 were slightly increased within 6 h, while they were dramatically decreased after longer treatment with LPS. However, the phosphorylation of 4EBP1 was constantly reduced (Figures 2A,F–H). Moreover, the Caco 2 cells were directly infected with *Salmonella* to figure out whether bacterial infection has a similar effect on the regulation of TFEF expression and functions as LPS. The results indicated that *Salmonella* also inhibited mTOR activation and increased TFEB, LAMP1, and LC3 (Figures 2I–O). To further demonstrate this upregulation of LAMP1 and LC3 by LPS and *Salmonella* infection, we used shRNA to silence the expression of TFEB in the Caco 2 cells and then treated these engineered cells with LPS or *Salmonella* infection. As expected, downregulating TFEB inhibited the induction of *LAMP1* and *MAP 1LC3B* (the gene for LC3) expression (Figure 2P). Additionally, gene set enrichment analysis (GSEA) was conducted, revealing that the autophagy- and lysosome-related genes were enriched after LPS stimulation in another colonic epithelial cell line, HT29 cells, as indicated by a Gene Expression Omnibus (GEO) database (Figures 2Q,R). Together, these observations suggest that LPS enhances TFEB and autophagy-lysosome protein expression, which is likely due to the inactivation of mTOR.

### LPS promotes the activity of TFEB in the epithelial cells

**Figure 3:**
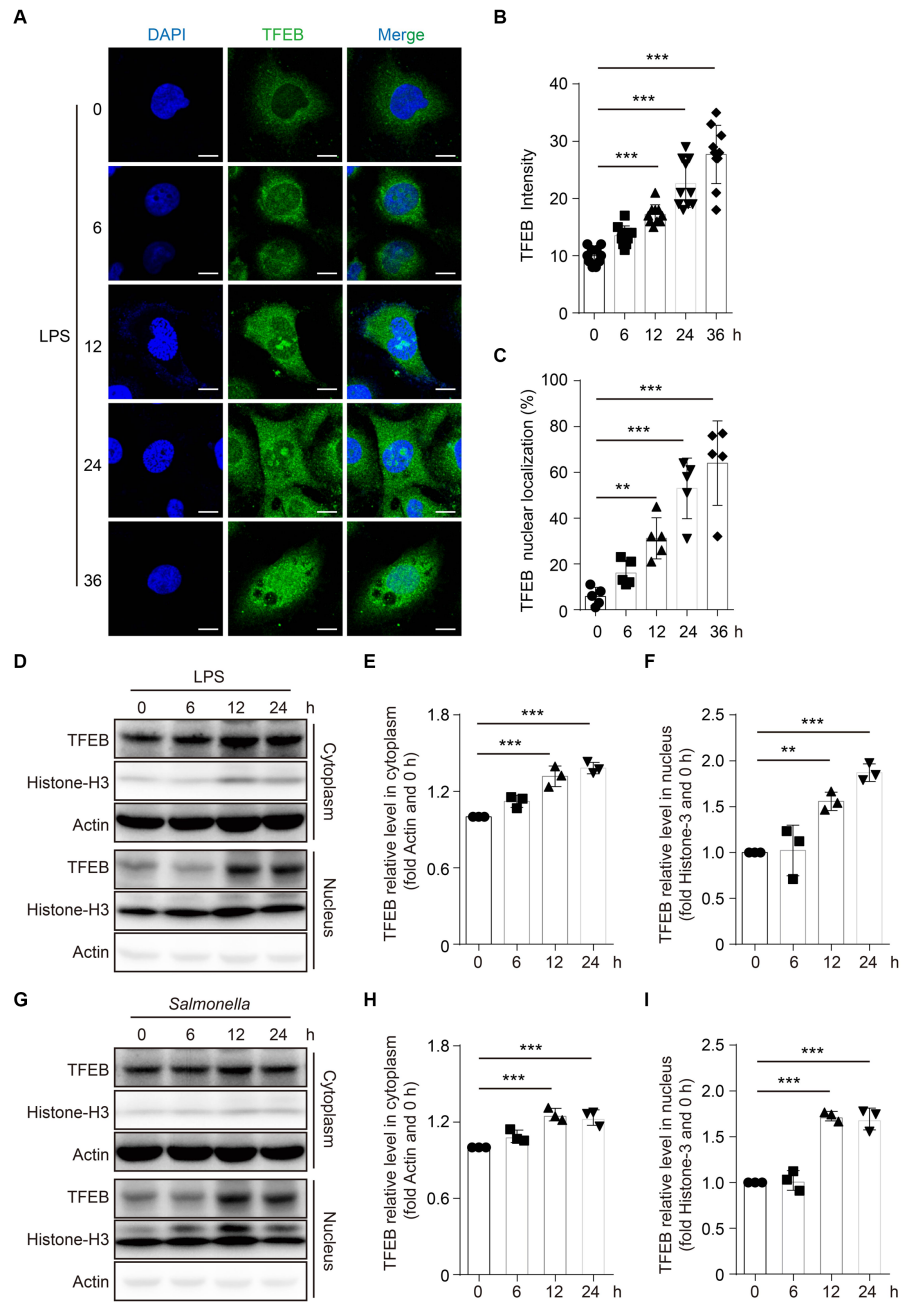
LPS promotes the TFEB to translocate into the nucleus. **(A–C)** Human colonic epithelium cell line Caco 2 cells were stimulated with 100 ng/mL of LPS for 0 h, 6 h, 12 h, 24 h, or 36 h. Immunofluorescence photos present the TFEB and DAPI staining in Caco 2 cells post-treatment at the indicated time **(A)**. Scale bars: 10 μm. The TFEB immunofluorescence intensity **(B)** and nuclear localization ratio **(C)** from random 10 cells of each group are quantified with ImageJ and presented as a histogram. **(D–F)** Caco 2 cells were stimulated with 1 μg/mL of LPS for 0 h, 6 h, 12 h, or 24 h. Cytoplasm and nucleus were separated. TFEB, histone 3, and actin were determined with Western blotting **(D)** and quantified with Image J **(E–F)**. Caco 2 cells were infected with *Salmonella* (MOI = 2) for 0 h, 6 h, 12 h, or 24 h. Cytoplasm and nucleus were separated. TFEB, histone 3, and actin were determined with Western blotting **(G)** and quantified with ImageJ **(H–I)**. Representative photos and bands are from three independent experiments. Mean values ± s.e.m. *p < 0.05, **p < 0.01, ***p < 0.001 using the one-way ANOVA with Dunnett’s test in **(B–C)**, **(E–F)**, and **(H–I)**.

As a TF, TFEB should be translocated to the nucleus to exert its transcriptional functions (Napolitano and Ballabio, 2016). To evaluate the alteration of TFEB activity after infection, TFEB and DAPI were determined in the Caco 2 cells following stimulation with LPS by immunofluorescence staining. The images showed that both the total fluorescence intensity of TFEB and the proportions of nuclear TFEB were significantly increased after the treatment with LPS (Figures 3A–C). To further confirm these observations, we determined the TFEB levels in the cytoplasm and nucleus of the Caco 2 cells after LPS stimulation. Consistently, we found significantly increased TFEB expression in both the cytoplasm and nucleus following the stimulation with LPS for 12 h and 24 h. However, there were no changes in TFEB levels within 6 h (Figures 3D–F). Furthermore, infection with *Salmonella* has a similar effect on the TFEB translocation (Figures 3G–I). Collectively, these results suggest that both LPS and *Salmonella* infections enhance the transcriptional activity of TFEB in the colonic epithelium.

### *Salmonella* facilitates TFEB translocation to the nucleus in mice colonic enterocytes *in vivo*

**Figure 4:**
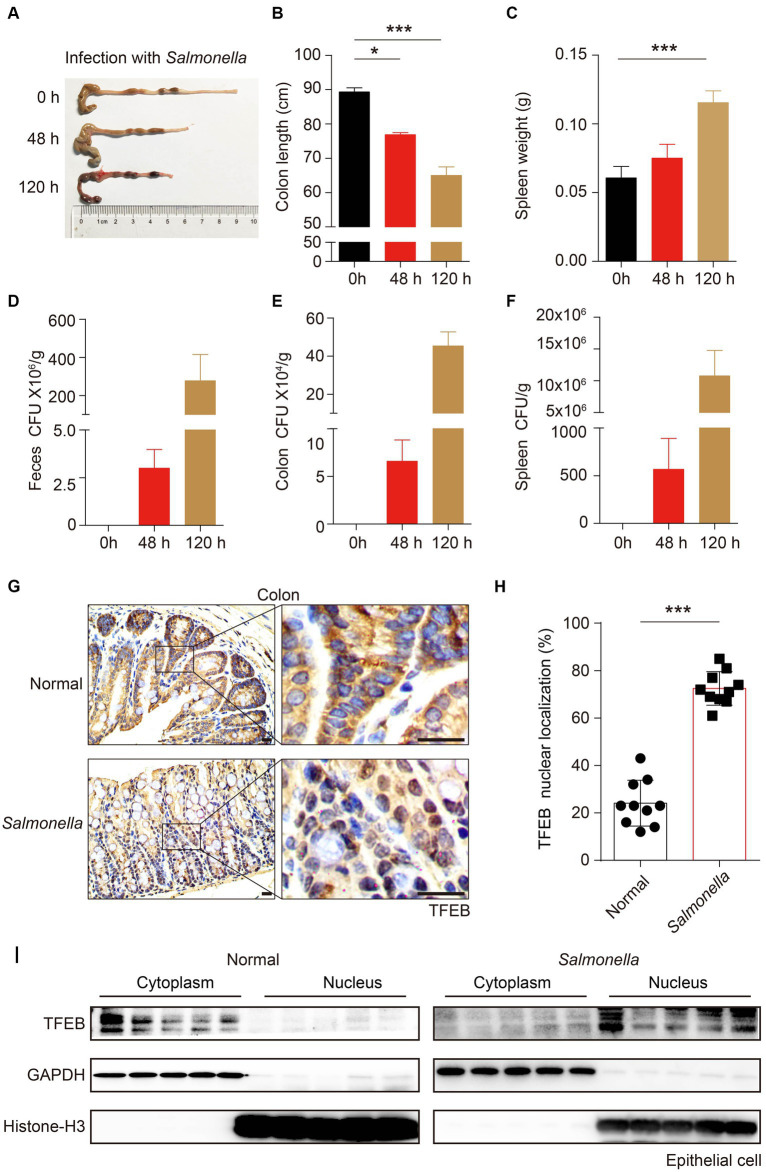
*Salmonella*-caused colitis presents as increased TFEB nuclear localization in the colonic epithelium. **(A–I)** C57/B6 mice were administered with or without 1 × 10^8^ *Salmonella* for 48 h or 120 h by oral gavage (5 mice per group). The length of the colon **(A–B)** and the weight of the spleen **(C)** were recorded, and the statistics are presented as a histogram. The number of bacteria (CFU) in mice feces **(D)**, colon **(E)**, and spleen **(F)** were recorded and the statistics are presented as a histogram. Colon tissues with infection or not were stained with TFEB **(G)**. The TFEB nuclear translocation was independently quantified and assessed by two pathologists without treatment information. Two fields of images were randomly selected in each histologic section, and the nuclear localization ratios were obtained by comparing the nuclear positive-staining cell number to the total cell number **(H)**. Determining the epithelial TFEB distribution in the cytoplasm and nucleus of normal colon tissue and *Salmonella*-infected colon tissue **(I)**. Scale bars: 20 μm. Mean values ± s.e.m.*p < 0.05; ***p < 0.001 using the one-way ANOVA with Dunnett’s test in **(B–C)** and Student’s *t*-test **(H)**.

To further substantiate that TFEB can be activated by infection *in vivo*, C57/B6 mice were infected with *Salmonella* (ampicillin-resistant) by oral gavage for 48 h or 120 h. After the indicated time, the mice were euthanized under anesthesia. The length of the colon was measured, and it showed a shrinkage of colon length in a time-dependent manner (Figures 4A,B). Additionally, the weight of the spleen also increased after infection (Figure 4C). Moreover, by culturing the tissue lysis solutions in LB plates containing ampicillin, we found a large number of *Salmonella* that survived in the feces, colon, and spleen of mice (Figures 4D–F). Thus, these observations confirmed that *Salmonella* caused colitis and invaded the colon of mice. Next, we sought to investigate whether the pathogenic bacteria modulated the activity of TFEB in the epithelium using immunohistochemistry (IHC) staining. The results showed a significant increase of the nuclear TFEB in the epithelium of these mice infected with *Salmonella* (Figures 4G,H). Moreover, we isolated the colonic epithelial cells and determined TFEB using Western blotting. In line with the histochemical analysis, *Salmonella* infection enhanced the nuclear translocation of TFEB (Figure 4I). Hence, these findings indicate that the *Salmonella* infection results in the activation of TFEB in colonic epithelial cells *in vivo*.

### LPS advances TFEB-mediated anti-bacterial response in the epithelial cells

**Figure 5:**
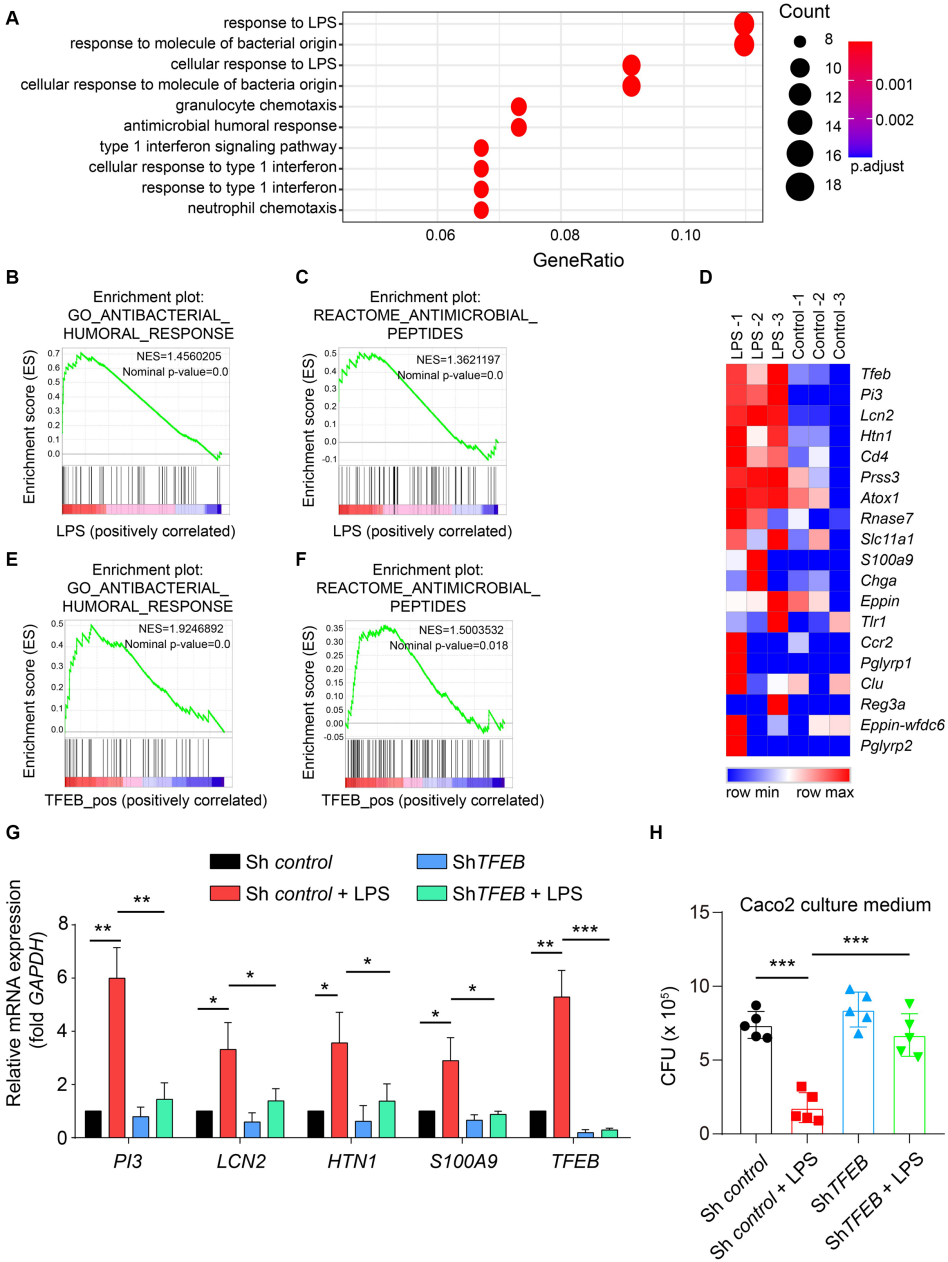
LPS boosts the TFEB-mediated anti-bacterial response in the epithelial cells. **(A)** GO annotation of 200 upregulated genes between LPS-treated and control HT29 cells. **(B)** Anti-bacterial response gene set, Z-score values, and enrichment plot after GSEA analysis between LPS-treated and control HT29 cells (NES = 1.4560205, *p* < 0.01, GSE113581). **(C)** Anti-microbial peptide gene set, Z-score values, and enrichment plot after GSEA analysis between LPS-treated and control HT29 cells (NES = 1.3621197, *p* < 0.01, GSE113581). **(D)** The heatmap shows the correlation between TFEB and anti-microbial peptide-related genes (GSE113581). **(E)** Anti-bacterial response gene set, Z-score values, and enrichment plot after GSEA analysis of 57 intestinal epithelial cell lines according to TFEB expression levels. (NES = 1.9246892, *p* < 0.01, CCLE). **(F)** Anti-microbial peptide gene set, Z-score values, and enrichment plot after GSEA analysis of 57 intestinal epithelial cell lines according to TFEB expression levels (NES = 1.5003532, *p* < 0.01, CCLE). **(G)** Histogram exhibits the relative mRNA expression of *PI3*, *LCN2*, *HTN1*, *S100A9*, and *TFEB* under the treatment of 100 ng/mL of LPS or not in sh*Control* or sh*TFEB* Caco 2 cells. The data were from three independent experiments. **(H)** Sh*Control* and sh*TFEB* Caco 2 cells were treated with 100 ng/mL of LPS for 48 h, and these cell culture media were collected to culture *Salmonella* for 12 h. The CFU of *Salmonella* was determined using the LB plates. Mean values ± s.e.m.*p < 0.05, **p < 0.01; ***p < 0.001 using the one-way ANOVA with Dunnett’s test in **(G–H)**. The permutation test was used in GSEA.

By clarifying the predominant distribution of TFEB in the epithelium and demonstrating its activation by LPS and *Salmonella in vitro* and in a mouse model, we subsequently aimed to determine the distinct functions executed by TFEB. Therefore, we used the GEO dataset and conducted a Gene Ontology (GO) analysis and found that most of the upregulated genes with LPS treatment were related to anti-bacterial response (Figure 5A). The GSEA of this dataset showed that post-administration with LPS, there was an enrichment of anti-bacterial response- and peptide-related genes (Figures 5B,C). Furthermore, the heatmap graph showed that LPS increased a large group of anti-bacterial peptides, such as *PI3*, *LCN2*, *HTN1*, and *S100A9*, that were positively related to TFEB (Figure 5D). To confirm the critical role of TFEB in bacterial defense, we utilized the Cancer Cell Line Encyclopedia (CCLE) gene set, which measured whole gene expression with RNA-seq in a series of cell lines and conducted GSEA. The results showed that genes linked to anti-bacterial response and peptides were enriched in TFEB highly expressed intestinal epithelial cell lines (Figures 5E,F). More importantly, the levels of several critical genes that encode anti-bacterial peptides, including *PI3*, *LCN2*, *HTN1*, and *S100A9*, were significantly increased after LPS treatment. However, these genes were dramatically decreased by the deficiency of *TFEB* (Figure 5G). To investigate whether these upregulated anti-bacterial peptides can suppress bacterial replication, we collected the supernatant of control Caco 2 and sh*TFEB* Caco 2 cells, with or without LPS treatment, and cultured *Salmonella*. After 12 h of culture, we found that fewer bacteria survived in the LPS-treated culture medium than in the non-treated medium, and this effect was diminished upon the reduction of TFEB expression (Figure 5H). In addition, to investigate other possible mechanisms that may contribute to the anti-bacterial response mediated by TFEB, we conducted GSEA to examine the relationship between TFEB and the tight junction and polarity of epithelial cells based on the CCLE dataset. The results revealed a less significant correlation between TFEB level and tight junction and polarity (Supplementary Figures S1A,B). We also tested the association between TFEB and inflammatory cytokine expression. GSEA and heatmaps showed that TFEB also did not affect the expression of cytokines such as IL-1, IL-6, and TNF in the epithelial cells (Supplementary Figures S1C,D). Therefore, epithelial TFEB contributes to the production of anti-bacterial peptides to fight infection.

## Discussion

Colitis is usually caused by infection (Azer and Sun, 2021), although the detailed interactions between the colon and pathogenic bacteria remain unknown. In our studies, we identified that LPS and *Salmonella* could activate TFEB located in colonic epithelial cells. The activated TFEB not only enhanced the autophagy-lysosome degradation pathway but also promoted the expression of the anti-bacterial peptide. Thus, we uncovered the key anti-bacterial role of TFEB in colonic enterocytes.

TFEB, one of the microphthalmia family of basic helix–loop–helix–leucine–zipper (bHLH-Zip) TFs (MiT family), was initially viewed as a key TF in controlling lysosomal gene expression and functions (Napolitano and Ballabio, 2016; He et al., 2020). A growing body of evidence has documented that it also regulates energy metabolism (Mansueto et al., 2017), cytokine production (Visvikis et al., 2014), and immune reshaping (Zhang et al., 2019). Moreover, its irreplaceable functions in the defense of bacteria are becoming increasingly recognized. Macrophages are known for their role in clearing invasive microbes as pioneers of innate immune cells, and the majority of research focused on the defensive role of TFEB in macrophages (Gray et al., 2016; El-Houjeiri et al., 2019; Rao et al., 2020; Schuster et al., 2022; Inpanathan et al., 2023). It is important to note that intestinal or colonic epithelial cells also function as the first defensive line. The detailed mechanisms of TFEB functioning in the colon need to be clarified. Our results demonstrated that TFEB was predominately expressed in the epithelial cells of the colon while being scarce in macrophages. Hence, the TFEB-mediated anti-bacterial responses should primarily occur in the epithelium, suggesting an accurate site for further reaches of TFEB in colitis or other colonic diseases.

LPS originating from damaged *Salmonella* or other Gram-negative pathogens is a risk factor present in the plasma of IBD patients (Caradonna et al., 2000). Although it has been reported that LPS may regulate the lysosome position in dendritic cells (Bretou et al., 2017) and macrophages (Mrakovic et al., 2012) and trigger TFEB nuclear localization in murine macrophages (El-Houjeiri et al., 2019), it remains unclear whether LPS affects the functions of TFEB in colonic epithelial cells. In this study, we verified that LPS enhanced the activity of TFEB and autophagy-lysosome molecule expression in human colonic epithelial cells. Interestingly, our previous findings showed that *Salmonella* suppressed TFEB expression and function in BMDM (Rao et al., 2020), whereas this study proved that *Salmonella* increased the nuclear TFEB ratio in colonic epithelium. Moreover, in addition to the autophagy-lysosome, TFEB participated in the regulation of anti-bacterial peptide expression rather than pro-inflammatory cytokine secretion, tight junction, and polarity of epithelial cells.

In conclusion, this study elucidated the precise localization of TFEB within the colon and examined the interplay between infection and epithelium facilitated by TFEB. These findings contribute to our understanding of colitis and offer potential advancements in infection management strategies.

## Materials and methods

### Reagents

The IHC antibody anti-TFEB (ab2636) was purchased from Abcam. Western-blot antibodies, including anti-LAMP1 (sc-20011, Santa Cruz Biotechnology), anti-LC3 (3,868, Cell Signaling Technology), anti-p-P65 (3,033, Cell Signaling Technology), anti-P65 (8,242, Cell Signaling Technology), anti-p-S6 (4,858, Cell Signaling Technology), anti-S6 (2,217, Cell Signaling Technology), anti-p-4EBP1 (2,855, Cell Signaling Technology), anti-4EBP1 (9,452, Cell Signaling Technology), anti-Actin (3,700, Cell Signaling Technology), anti-Histone H3 (ab1791, Abcam), donkey Anti-Goat IgG H&L (FITC, ab6881), and goat anti-rabbit IgG H&L (HRP, ab6721) were purchased from Abcam. DAPI (d9542) and LPS (L6386) were purchased from Sigma Aldrich.

### Cell culture and stimulation

The Caco 2 cells (SCSP-5027) were purchased from the National Collection of Authenticated Cell Cultures. The cells were cultured with DMEM (10,566,016, Thermo Fisher Scientific) containing 10% FBS, streptomycin, and penicillin at 37°C, 5% CO_2_. The Caco 2 cells were planted onto six-well plates and stimulated with 100 ng/mL of LPS for 0 h, 6 h, 12 h, 24 h, or 36 h. The cells were collected for Western blotting or immunofluorescence staining. BMDMs were differentiated and cultured as previously described (Xia et al., 2019).

### Western blotting

The LPS-treated Caco 2 cells, BMDMs, primary murine colonic epithelial cells, colon tissue without epithelium, and integrated colon tissue were lysed with RIPA buffer containing proteases and phosphatase inhibitor cocktail (78,440, Thermo Fisher Scientific). The concentration of cell lysate was measured using a BCA kit (A53226, Thermo Fisher Scientific). After adding 5 × protein loading buffer and boiling for 5 min, the protein samples were loaded onto a 10% SDS-PAGE gel. Separated proteins were transferred onto a PVDF membrane post-electrophoresis for 2 h. The PVDF membrane containing proteins was blocked with 5% BSA and incubated with primary antibodies overnight. The blots were visualized using electrochemiluminescence (ECL) after incubation with HRP-conjugated second antibody. Quantification was performed using Image J.

### IHC staining

Infected or non-infected murine colonic tissues were fixed with formalin and embedded in paraffin. The tissues were sectioned into slices and then baked for 1 h before being dewaxed with dimethylbenzene. Dehydration was performed using ethanol and endogenous peroxidase was eliminated with 3% H_2_O_2_. The sections were incubated with a 5% BSA and then an anti-TFEB antibody (1:200). After staining with an HRP-conjugated second antibody, the sections were visualized with diaminobenzidin (DAB) and hematoxylin. An Olympus microscope was used to capture images.

### Immunofluorescence staining

The LPS-treated Caco 2 cells were fixed with 4% paraformaldehyde for 20 min. The cell membrane was permeated with 0.5% Triton X-100 after being washed with PBS. The cell slides were blocked with a 5% BSA before being incubated with anti-TFEB. The slides were stained with FITC-conjugated secondary antibody and DAPI and then photographed using confocal microscopy (Zeiss, Germany).

### Cell cytoplasm and nucleus extraction

To separate the cell cytoplasm and nucleus, a commercial kit (P0028, Beyotime) was used, and the detailed experiments were conducted according to the manufacturer’s instructions.

### Quantitative RT-PCR

The total RNA was isolated using TRIZOL (15,596,026, Invitrogen™), and the cDNA was obtained using a commercial kit (4,374,967, Applied Biosystems™). Next, the qRT-PCR was performed using SYBR green (A46110, Applied Biosystems™), and the primers used are as follows:

*TFEB*, F-CCTGGAGATGACCAACAAGCAG; R-TAGGCAGCTCCTGCTTCACCAC.

*PI3*, F-CGCTGCTTGAAAGATACTGACTG; R-ACGGCACAGGTGCAGCAAGGA.

*LCN2*, F-GTGAGCACCAACTACAACCAGC; R-GTTCCGAAGTCAGCTCCTTGGT.

*HTN1*, F-CATCATGGGTATAGAAGAAAATTCC; R-TGCCCCATGATTACTAAGGATATC.

*S100A9*, F-GCACCCAGACACCCTGAACCA; R-TGTGTCCAGGTCCTCCATGATG.

*GAPDH*, F-GTCTCCTCTGACTTCAACAGCG; R-ACCACCCTGTTGCTGTAGCCAA.

*LAMP1*, F-GGCCTCTTGCGTCTGGTAAC; R-AAAGGTACGCCTGGATGGTG.

*MAP1LC3B*, F-GAGAAGCAGCTTCCTGTTCTGG; and R-GTGTCCGTTCACCAACAGGAAG.

shRNA lentivirus construction and shTFEB Caco 2 cells generation.

The scrambled shRNA lentivirus target human TFEB was constructed using pLVX-shRNA2 plasmids (Clontech Laboratories, Inc., 632,179), following the procedure described previously (15). These plasmids were transfected onto 293 T cells to generate lentivirus in the supernatant. For the generation of sh*TFEB* Caco 2 cells, the epithelial cells were infected with lentivirus for approximately 5 days, and the TFEB level was determined by RT-PCR. The shRNA sequences are as follows:

Forward: GATCCCCACTTTGGTGCTAATAGCTTTCAAGAGAAGCTATTAGCACCAAAGT.

GGGTTTTTG;

Reverse: AATTCAAAAACCCACTTTGGTGCTAATAGCTTCTCTTGAA AGCTATTAGCAC.

CAAAGTGGG.

### Mouse model

*Salmonella* (SL1344, ampicillin-resistant) was a gift from Xiang-ping Yang lab, and the protocol for culturing bacteria was the same as before (Rao et al., 2020). For the infection *in vivo*, *Salmonella* was cultured in 3 mL of LB medium for 4 h and measured using a spectrophotometer (Thermo Fisher Scientific). The infection model was performed as previously described (Xia et al., 2019) and in accordance with Institutional Animal Care guidelines. In brief, 30 C57/B6 mice were randomly arranged into three groups (5 mice per group). Two groups of mice were intragastrically infected with 1 × 10^8^ of *Salmonella* for 48 h or 120 h. Infected mice or healthy mice were euthanized under anesthesia. The length of the colon and the weight of the spleen were determined. The tissues were homogenized and resuspended in sterile PBS. After serial dilution, the suspensions were plated onto LB plates with ampicillin. The number of bacterial colonies was quantified.

### Mouse colonic epithelial cell extraction

For the extraction of colonic epithelial cells, normal or *Salmonella*-infected colon tissues of C56/B6 mice were collected from the mouse model (5 mice per group). After three washes with sterile PBS, the colonic lumens were injected and filled with trypsin (Thermo Fisher Scientific, 25,200,072). The two ends of the colon lumen were sealed with surgical thread ties and incubated in a cell incubator for 1 h at 37°C. After digestion, these epithelial cells in the colonic lumen could be pipetted and ejected. Then, a 10% FBS was added to the cell suspensions, and the colonic epithelial cells and non-epithelial cell colon tissues were obtained. After centrifugation and two washes with ice-cold PBS, these newly isolated epithelial cells were cultured in DMEM (10% FBS) for approximately 30–60 min. The suspended epithelial cells in the culture medium were then collected for further determination. The purity of epithelial cells was determined by flow cytometry using EpCAM antibodies (Biolegend, 118,207), a marker of epithelial cells.

### Bacteria survive in culture medium

The control Caco 2 cells and sh*TFEB* Caco 2 cells were treated with LPS (100 ng/mL) for 48 h, and the culture media were collected and centrifuged at 10000 rpm. The supernatants were used to culture *Salmonella* in a concentration of 5 × 10^5^/mL. After 12 h of culture, the media containing *Salmonella* were collected and plated on LB plates at an appropriate dilution. The CFU of *Salmonella* was recorded and quantified.

### Data collection

The gene expression profile of HT29 cells was downloaded from the GEO database, specifically from the GSE113581 dataset.^1^ HT29 cells were treated with LPS or vehicle (DMSO). We downloaded and processed the RNA-seq data of 57 intestinal epithelial cell lines from the CCLE.^2^

### Bioinformatic analysis

We downloaded related gene sets from the Molecular Signatures of GSEA official website^3^ and used GSEA software to determine the different pathways related to the target genes. The GO annotation for the targeted genes was performed using the R packages “clusterProfiler” and “enrichplot” to determine the upregulated genes in epithelial cells following LPS treatment. The CCLE was used to interrogate the transcriptomics data of the inflammatory cytokine-related genes in a panel of 57 intestinal epithelial cell lines (see text footnote 2, respectively).

### Statistical analysis

Statistical analyses were performed using GraphPad Prism 8. An unpaired two-tailed Student’s *t*-test was used to compare two groups, while a one-way ANOVA with Dunnett’s test was used to compare at least three groups. Results were presented as mean values ± standard error of the mean (s.e.m). A *p*-value less than 0.05 was considered significant.

R (version 4.0.4) was used to carry out differential analysis of the RNA expression profile, and the “limma” package was used to standardize and analyze all data. Differential genes were screened based on corrected *p*-values less than 0.05 and absolute log fold change greater than 1.

## Data availability statement

The original contributions presented in the study are included in the article/Supplementary material, further inquiries can be directed to the corresponding author/s.

## Ethics statement

The animal studies were approved by the Animal Care and Use Committee of Tongji Medical College, Huazhong University of Science and Technology (Wuhan, 3275).

## Author contributions

SR: Conceptualization, Writing – original draft, Data curation, Investigation, Methodology, Project administration. PH: Investigation, Project administration, Formal analysis, Writing – review & editing. Y-YQ: Investigation, Project administration, Software, Writing – review & editing. YX: Conceptualization, Writing – original draft. HZ: Supervision, Writing – review & editing.

## Footnotes

^1^ http://www.ncbi.nlm.nih.gov/geo/

^2^ https://portals.broadinstitute.org/ccle/

^3^ https://software.broadinstitue.org/gsea/
