# Supplementary material for: Colonic epithelial cell-specific TFEB activation: a key mechanism promoting anti-bacterial defense in response to Salmonella infection
Source: Front Microbiol. 2024 Apr 22;15:1369471. doi: 10.3389/fmicb.2024.1369471 (PMC11070474; doi:10.3389/fmicb.2024.1369471)

**Colonic Epithelial Cell-Specific TFEB Activation: A Key Mechanism Promoting Anti-Bacterial Defense in Response to Salmonella Infection**

Shanshan Rao^1^, Pu Huang^2^, Yi-Yu Qian^3,4^, Yu Xia^3,4#^, Hongfeng Zhang^1#^

^1^Department of Pathology, the Central Hospital of Wuhan, Tongji Medical College, Huazhong University of Science and Technology, Wuhan, China.

^2^Department of Obstetrics and Gynecology, Shandong Provincial Hospital Affiliated to Shandong First Medical University, Jinan, People's Republic of China.

^3^Cancer Biology Research Center (Key Laboratory of the Ministry of Education, Hubei Provincial Key Laboratory of Tumor Invasion and Metastasis), Tongji Hospital, Tongji Medical College, Huazhong University of Science and Technology, Wuhan, China.

^4^National Clinical Research Center for Obstetrics and Gynecology, Department of Gynecological Oncology, Tongji Hospital, Tongji Medical College, Huazhong University of Science and Technology, Wuhan, China.

Corresponding Author #:

Hongfeng Zhang, Department of Pathology, the Central Hospital of Wuhan, Tongji Medical College, Huazhong University of Science and Technology, Wuhan, China. E-mail: zhf152@163.com.

Yu Xia, Cancer Biology Research Center (Key Laboratory of the Ministry of Education, Hubei Provincial Key Laboratory of Tumor Invasion and Metastasis), Tongji Hospital, Tongji Medical College, Huazhong University of Science and Technology, Wuhan, China. E-mail: xiayu_hb@sina.com.

**Figure S1. TFEB is irrelevant with the tight junction, polarity, and cytokine expression in intestinal epithelial cells**. (A) Tight junction gene set, Z-score values, and enrichment plot after GSEA analysis of 57 intestinal epithelial cell lines according to TFEB expression levels. (NES = 1.557084, P = 0.190). (B) Cell polarity gene set, Z-score values, and enrichment plot after GSEA analysis of 57 intestinal epithelial cell lines according to TFEB expression levels. (NES = -0.8630512, P = 0.725). (C) Inflammatory cytokine gene set, Z-score values, and enrichment plot after GSEA analysis of 57 intestinal epithelial cell lines according to TFEB expression levels. (NES = -1.3343972, P = 0.135). (D) The heat map exhibits the correlation between TFEB and inflammatory cytokine-related genes. The permutation test was used in GSEA.


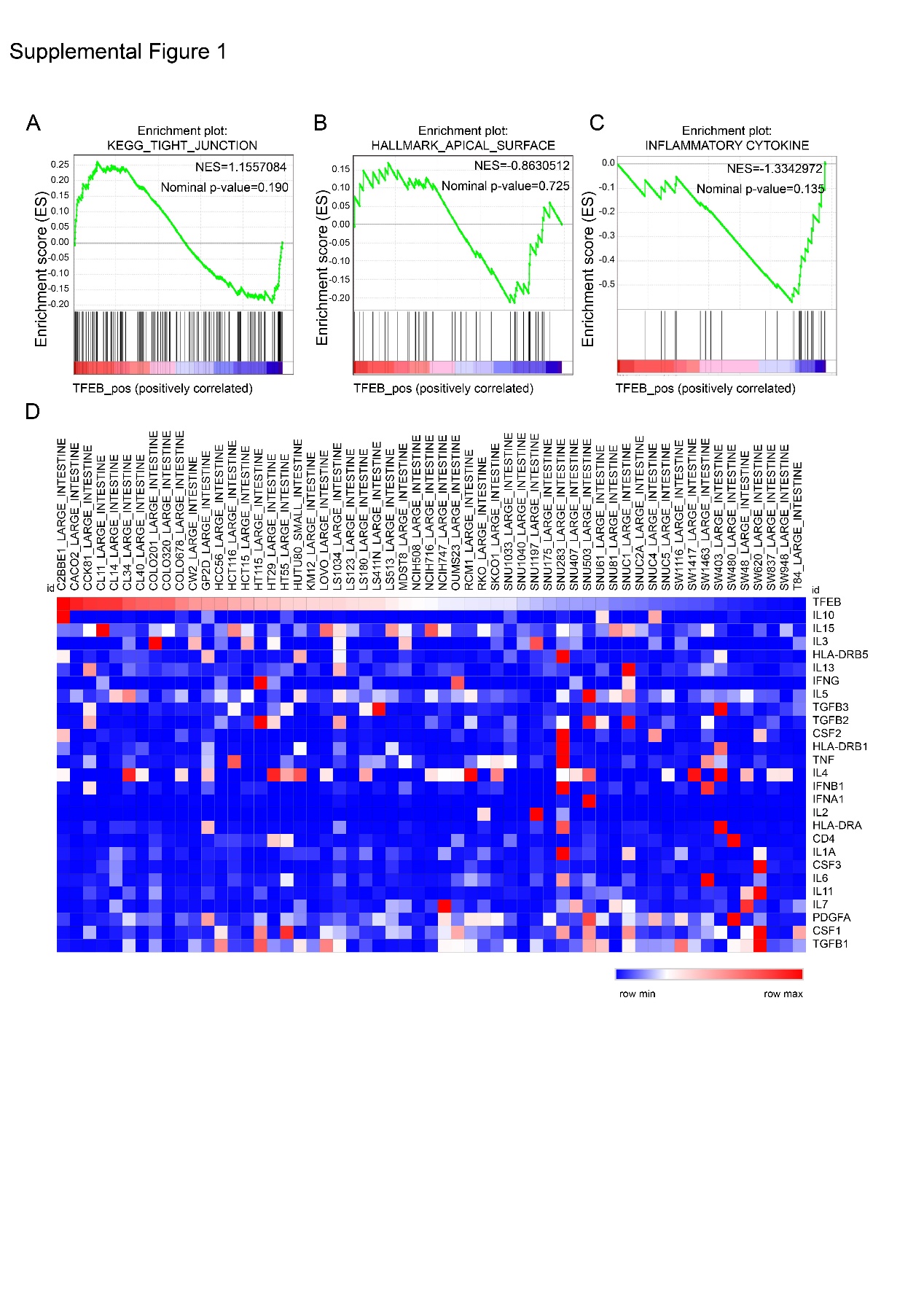

Supplement: Supplementary file 1 [file Table_1.docx]
